# Preoperative ultrasound and FNA in the diagnosis of axillary involvement in invasive breast cancer: correlation with intraoperative one-stop nucleic acid amplification and final histology

**DOI:** 10.1186/bcr3305

**Published:** 2012-11-09

**Authors:** EK Jackson, N Howes, A Jones, Z Rayter, A Valencia

**Affiliations:** 1 Bristol Royal Infirmary, Bristol, UK

## Objective

To assess our preoperative pickup of malignant axillary lymph nodes by ultrasound and FNA compared with one-stop nucleic acid amplification (OSNA) and final histology.

## Methods

At our unit all patients with invasive breast cancer undergo axillary ultrasound, and those with suspicious or equivocal findings undergo axillary FNA. If FNA is positive we proceed to axillary node clearance (ANC). If axillary assessment is normal we perform sentinel node biopsy (SNB) with intraoperative OSNA, unless planned for neoadjuvant chemotherapy or primary medical therapy. In patients with positive OSNA, further surgery is performed as per hospital protocol. Retrospective correlation of preoperative axillary ultrasound and FNA findings with intraoperative OSNA in the SNB group and final histology in the ANC group was performed, with patients diagnosed with invasive breast carcinoma between September 2010 and September 2011.

## Results

**Figure 1:**
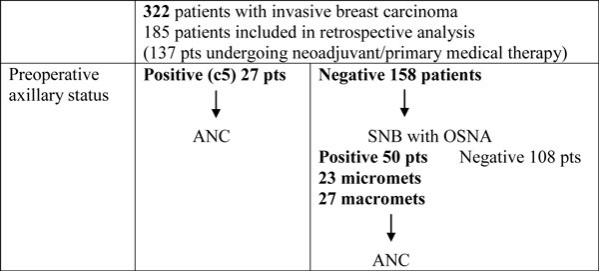


See Figure 1. Seventy-seven patients were lymph node-positive (LN+ve) overall (77/185 = 42% population LN+ve). Twenty-seven patients had preoperative LN+ve diagnosis with USS and FNA (27/77 = 35% of LN+ve patients have preoperative diagnosis). Imaging of the OSNA macromet subset is reviewed and examples discussed (*n *= 27: 22 imaging normal, three equivocal, two abnormal).

## Conclusion

This is the first study correlating preoperative imaging with OSNA. Our high rate of preoperative diagnosis is encouraging, but suggestions for improvement are discussed.

